# Práticas clínicas relacionadas a cânulas nasais de alto fluxo em terapia intensiva pediátrica no Brasil em comparação com as de outros países: um inquérito brasileiro

**DOI:** 10.5935/0103-507X.20210055

**Published:** 2021

**Authors:** José Colleti Júnior, Atsushi Kawaguchi, Orlei Ribeiro de Araujo, Daniel Garros

**Affiliations:** 1 Unidade de Terapia Intensiva Pediátrica, Hospital Santa Catarina - São Paulo (SP), Brasil.; 2 Departamento de Pediatria, Universidade de Montreal, CHU Sainte-Justine - Montreal, QC, Canadá.; 3 Grupo de Apoio ao Adolescente e à Criança com Câncer, Instituto de Oncologia Pediátrica, Universidade Federal de São Paulo - São Paulo (SP), Brasil.; 4 Departamento de Pediatria, Division of Critical Care, Stollery Children’s Hospital, University of Alberta - Edmonton, AB, Canadá.

**Keywords:** Cânula, Oxigenoterapia, Cuidados críticos, Ventilação não invasiva, Insuficiência respiratória, Unidades de terapia intensiva pediátrica, Inquéritos e questionários, Brasil, Estados Unidos, Canadá, Reino Unido, Índia

## Abstract

**Objetivo:**

Descrever as práticas clínicas atuais relacionadas à utilização de cânula nasal de alto fluxo por intensivistas pediátricos brasileiros e compará-las com as de outros países.

**Métodos:**

Para o estudo principal, foi administrado um questionário a intensivistas pediátricos em países das Américas do Norte e do Sul, Ásia, Europa e Austrália/Nova Zelândia. Comparou-se a coorte brasileira com coortes dos Estados Unidos, Canadá, Reino Unido e Índia.

**Resultados:**

Responderam ao questionário 501 médicos, dos quais 127 eram do Brasil. Apenas 63,8% dos participantes brasileiros tinham disponibilidade de cânula nasal de alto fluxo, em contraste com 100% dos participantes no Reino Unido, no Canadá e nos Estados Unidos. Coube ao médico responsável a decisão de iniciar a utilização de uma cânula nasal de alto fluxo segundo responderam 61,2% dos brasileiros, 95,5% dos localizados no Reino Unido, 96,6% dos participantes dos Estados Unidos, 96,8% dos médicos canadenses e 84,7% dos participantes da Índia; 62% dos participantes do Brasil, 96,3% do Reino Unido, 96,6% dos Estados Unidos, 96,8% do Canadá e 84,7% da Índia relataram que o médico responsável era quem definia o desmame ou modificava as regulagens da cânula nasal de alto fluxo. Quando ocorreu falha da cânula nasal de alto fluxo por desconforto respiratório ou insuficiência respiratória, 82% dos participantes do Brasil considerariam uma tentativa com ventilação não invasiva antes da intubação endotraqueal, em comparação com 93% do Reino Unido, 88% dos Estados Unidos, 91,5% do Canadá e 76,8% da Índia. Mais intensivistas brasileiros (6,5%) do que do Reino Unido, Estados Unidos e Índia (1,6% para todos) afirmaram utilizar sedativos com frequência concomitantemente à cânula nasal de alto fluxo.

**Conclusão:**

A disponibilidade de cânulas nasais de alto fluxo no Brasil ainda não é difundida. Há algumas divergências nas práticas clínicas entre intensivistas brasileiros e seus colegas estrangeiros, principalmente nos processos e nas tomadas de decisão relacionados a iniciar e desmamar o tratamento com cânula nasal de alto fluxo.

## INTRODUCTION

High-flow nasal cannula (HFNC) therapy is a relatively new noninvasive ventilation (NIV) therapy that seems to be well tolerated in children.^(1,2)^ High-flow nasal cannulas have been used for many different purposes, ranging from first-line therapy for children with acute viral bronchiolitis, mild acute respiratory distress syndrome, and pneumonia to postextubation failure prevention.^(1,3)^ Although the physiological mechanisms of HFNC are still unknown, it is thought that HFNC supports respiration by reducing work of breathing,^(4-6)^ decreasing patient energy expenditure by providing heated and humidified inhaled gas, improving lung compliance, decreasing dead space, and increasing lung mucociliary clearance.^(7,8)^

There are no widely accepted guidelines regarding best clinical practices related to the use of HFNC therapy. A lack of evidence may also lead to significant variations in clinical criteria for starting, weaning, and discontinuing this form of respiratory support, which raises concerns about delays in needed escalation and associated morbidity as well as increases in the length of hospital stay in patients given HFNC therapy. A recently published survey among pediatric intensivists revealed important differences in daily practices around the world.^(9)^ There are no data regarding the availability of HFNCs and the clinical practices of Brazilian pediatric intensivists related to HFNC therapy.

This study aimed to describe current clinical practices related to the use of HFNC therapy by Brazilian pediatric intensivists and compare them with those of intensivists from other countries as a subset analysis of a larger worldwide survey.^(9^

## METHODS

This study was a *post hoc* subgroup analysis of data collected from a survey conducted in collaboration with several regional pediatric critical care societies.^(9)^ A cross-sectional questionnaire was administered to pediatric intensive care unit (ICU) physicians practicing in North and South America, Asia, Europe, and Australia/New Zealand. The survey construction process and survey characteristics are described elsewhere.^(9)^ The survey was approved by the Health Research Ethics Board of the University of Alberta, Canada, and the *Hospital Santa Catarina*, São Paulo (SP), Brazil. The survey was also approved by AMIBnet, which is the research branch of the *Associação de Medicina Intensiva Brasileira* (AMIB). The survey was distributed via email to pediatric intensivists registered with the AMIB in October 2018. A second email was sent 2 months later to increase the number of respondents.

We performed a secondary analysis of the consolidated data to compare practices in Brazil, the United Kingdom, India, the United States, and Canada. These countries were chosen because they had comparable sample sizes in the survey. We choose to compare the Brazilian cohort with cohorts in North America (the United States and Canada) and the United Kingdom as representative high-income countries (HICs) and with a cohort in India as a country with a similar economic background as Brazil. These countries also had comparable sample sizes in the survey.

The questionnaire enquired about the characteristics of intensivists and hospitals, HFNC practices, supportive treatments, and HFNC research, and specific questions for each of these domains were created. The final version of the survey was developed using REDCap with the appropriate safeguards for confidentiality. We included all attending pediatric ICU physicians actively working at the time of the survey.

Descriptive data are expressed as the proportion (%) of respondents. To compare proportions between pairs of responses, we used Pearson’s chi-squared test with Yates’ continuity correction. When multiple responses were given, we used the Marascuilo procedure to simultaneously test the differences between all pairs of proportions. All statistical tests were performed with R software, version 3.6.1 (The R Foundation for Statistical Computing, 2019).

## RESULTS

We analyzed 501 responses of intensivists from five countries (Brazil, 127 respondents; United Kingdom, 81 respondents; United States, 146 respondents; Canada, 62 respondents; India, 85 respondents). The response rate of Brazilian intensivists was 44.8%.

**Figure 1:**
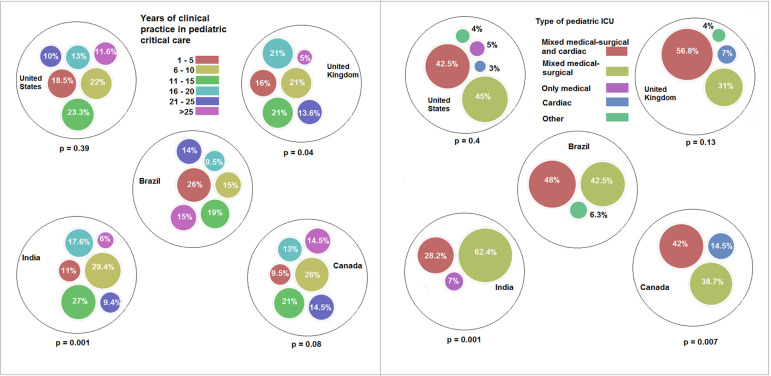
Years of experience in pediatric intensive care and type of pediatric intensive care unit. ICU - intensive care unit. P-values refer to comparisons between Brazil and each one of the countries.

**Figure 2:**
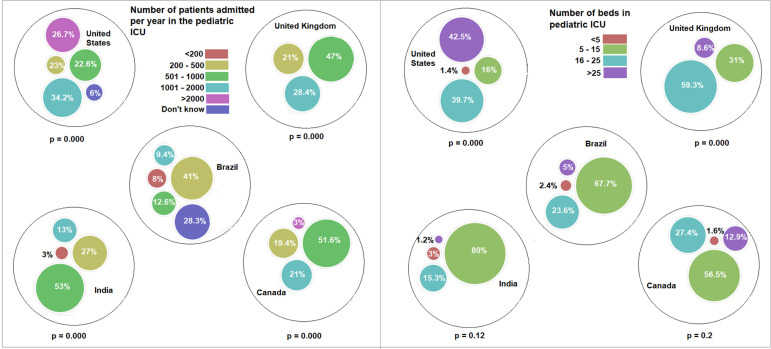
Patients admitted per year and beds in the pediatric intensive care unit. ICU - intensive care unit. P-values refer to comparisons between Brazil and each one of the countries.

The respondents from Brazil had fewer years of clinical practice than those from the other countries, and in Brazil, pediatric ICUs were predominantly mixed units (medical-surgical and medical-surgical and cardiac, 90.5%); this proportion was similar in the United States and the United Kingdom but different in Canada and India (Figure 1). The number of pediatric ICU beds per unit significantly differed between Brazil and the United Kingdom and between Brazil and the United States: in both the United Kingdom and the United States, the largest proportion of pediatric ICUs had more than 16 beds. Twenty-eight percent of the respondents in Brazil did not know the number of admissions per year to their pediatric ICUs, whereas 53.6% of the ICUs admitted between 200 and 1000 patients per year (Figure 2).

Academic profile also differed in Brazil, where 52.8% of the respondents said their hospitals were affiliated with a university, *versus* 96% in the United Kingdom, 92.5% in the United States, 98.4% in Canada (p < 0.001), and 65.9% in India (p = 0.002).

Regarding the types of respiratory support available, only 63.8% of respondents in Brazil had an HFNC available in their institutions, in contrast to 100% of respondents from the United Kingdom, Canada, and the United States and 97.6% in India (p < 0.001)). The proportions were similar regarding the availability of NIV (94.5% in Brazil, 98.8% in the United Kingdom, 98.6% in the United States, 98.4% in Canada, and 95.3% in India) (p ≥ 0.1). High-frequency oscillatory ventilation (HFOV) was available to 53.3% of the respondents in Brazil, 95% in the United Kingdom, 97.3% in the United States, 96.8% in Canada, and 80% in India (p < 0.001). Extracorporeal membrane oxygenation was available to 24.4% of the respondents in Brazil, 66.7% in the United Kingdom, 56.3% in the United States, 75.8% in Canada, and 48.2% in India (p < 0.001).

### Decisions and applications related to high-flow nasal cannula therapy

The attending intensivists were responsible for the decision to start HFNC therapy according to 61.2% of the respondents in Brazil compared to 95% of respondents in the United Kingdom, 96.6% in the United States, 96.8% in Canada, and 84.7% in India (p < 0.001). Respiratory therapists were appointed according to 25% of respondents in Brazil, 4% in the United Kingdom (p < 0.001), 21% in the United States (p = 0.5), 37% in Canada (p = 0.06), and 1% in India (p < 0.001).

A total of 62% of the respondents in Brazil said that the attending intensivists were responsible for the decision to wean or modify HFNC settings (Table 1). The decision was much less frequently made by trainees such as fellows in Brazil (8.7%), whereas it was made by trainees according to 86.4% of respondents in the United Kingdom, 82.2% in the United States, 75.8% in Canada, and 52.9% in India (p < 0.001 for all).

**Table 1:** Clinical markers used to assess the efficacy of high-flow nasal cannula and to guide weaning

	Brazil	United Kingdom	United States	Canada	India
Which clinical markers would you mainly use to decide that HFNC was not working for patients with a primary respiratory disease (e.g., bronchiolitis, pneumonia)?					
Need to increase FiO_2_ to > 0.60 (needing over 60% O_2_)	41.7	63.0*	56.8*	66.1†	69.4†
Worsening respiratory acidosis with PaCO_2_ > 60mmHg or > 8kPa	36.2	65.4†	74.7†	77.4†	62.4†
Significantly increased work of breathing or lack of improvement in severe respiratory distress	48.0	76.5†	85.6†	93.5†	72.9†
Significantly increased heart rate or lack of improvement in severe tachycardia	39.4	65.4†	51.4‡	48.4‡	58.8*
Significantly increased respiratory rate or lack of improvement in severe tachypnea	43.3	70.4†	78.8†	88.7†	71.8†
Development of apneas requiring intermittent mild stimulation	41.7	67.9†	71.2†	71.0†	49.4‡
Worsening of scores on a scoring system (like the Wood-Downes scale)	13.4	13.6	15.8‡	6.5‡	8.2‡
What makes you decide to wean from HFNC?					
Improvement in respiratory distress	46.5	76.5†	82.9†	93.5†	71.8†
Improvement in heart rate	25.2	64.2†	39.7*	32.3‡	43.5*
Improvement in respiratory rate	37.0	75.3†	76.7†	83.9†	64.7†
Improvement in oxygenation	39.4	77.8†	65.8†	82.3†	67.1†
Improvement in scores according to a scoring system	13.4	6.2‡	17.8‡	4.8*	1.2†

HFNC - high-flow nasal cannula; FiO2 - fraction of inspired oxygen; PaCO_2_ - partial pressure of carbon dioxide. The sum is not 100%, as some questions were not answered. * p < 0.05; † p ≤ 0.001; ‡ p ≥ 0.05 in comparison to Brazil. Results expresses as %.

Only 3.1% of the Brazilian respondents reported that HFNC therapy was used in general wards, whereas 56.8% of respondents in the United Kingdom, 59.6% in the United States, 46.8% in Canada, and 17.6% in India (p < 0.001 for all) reported HFNC therapy use in general wards. HFNC therapy was used in high-dependency care or pediatric ICU step-down units according to 5.5% of respondents in Brazil, 70.4% in the United Kingdom, 26% in the United States, 19.4% in Canada, and 24.7% in India (p < 0.001 for all). The proportions of respondents that reported the use of HFNC therapy in in the emergency department were 22% in Brazil, 38.3% in the United Kingdom (p = 0.01), 56.8% in the United States (p < 0.001), 42% in Canada, (p = 0.002) and 9.4% in India (p = 0.01). A total of 11.8% of respondents in Brazil reported the use of HFNC therapy in the neonatal ICU in Brazil compared to 37% of respondents in the United Kingdom, 48.6% in the United States, 40.3% in Canada, and 30.6% in India (p < 0.001 for all).

The responses regarding clinical indications, diagnoses, cannula size, and the existence of a written policy or protocol are summarized in table 2.

**Table 2:** Responses regarding clinical indications, diagnoses, size of the cannula, and the existence of a written protocol

	Brazil	United Kingdom	United States	Canada	India
For which patient diagnoses is HFNC used in the pediatric ICU in your personal practice?					
Postextubation	46.5	81.5*	86.3 *	79.0*	71.8*
Bronchiolitis	57.5	87.7*	92.5*	93.5*	87.5*
Asthma	45.0	68.0†	67.8†	69.4*	58.8‡
Pneumonia	32.3	82.7*	90.4*	88.7*	73*
Parenchymal lung disease other than pneumonia	21.3	76.6*	84.2*	75.8*	56.5*
Upper airway obstruction (such as croup)	28.3	37.0‡	63.0*	51.6*	44.7†
Cardiac failure	23.6	69.0*	57.5*	69.4*	57.5*
Neuromuscular weakness	11.8	65.4*	52.7*	51.6*	31.8*
For which clinical indications would you consider starting HFNC in the pediatric ICU in your personal practice					
Hypoxia	48.8	81.5*	84.9*	90.3*	68.2†
Respiratory acidosis	22.8	60.5*	63*	69.4*	32.9‡
Respiratory distress or increased work of breathing	44.9	90.0*	89.7*	93.5*	82.4*
Routinely after extubation	20.5	14.6‡	22.1‡	9.7‡	25.9‡
Support for heart failure	20.5	63.0*	47.9*	58.0*	49.4*
Routinely after removing NIV	17.3	17.3‡	32.9†	12.9‡	20.0‡
How do you determine the HFNC size in your personal practice?					
Strictly follow manufacturer order/criteria	44.0	43.2	26.7	38.7	69.4
Follow local guidelines (if different)	7.0	19.8	19.2	12.9	4.7
No specific criteria used	0.8	6.2	12.3	11.3	7
Only one size available	0.8	-	1.4	-	2.4
Do not know	3.9	11.0	24.7	25.8	-
p-value for multiple proportions		0.002	0.000	0.000	0.05
Do you have a written guideline/policy/protocol on how to start and how to wean HFNC in your pediatric ICU?					
Yes	30.7	45.7‡	23.3†	25.8†	16.5*

HFNC - high flow nasal cannula; ICU - intensive care unit; NIV - noninvasive ventilation. Note: the sum is not 100%, as some questions were not answered. * p ≤ 0.001; † p < 0.05; ‡ p ≥ 0.05 in comparison to Brazil. Results expressed as % if not indicated in a different way.

### Clinical scenarios

#### Case 1

**Figure 3:**
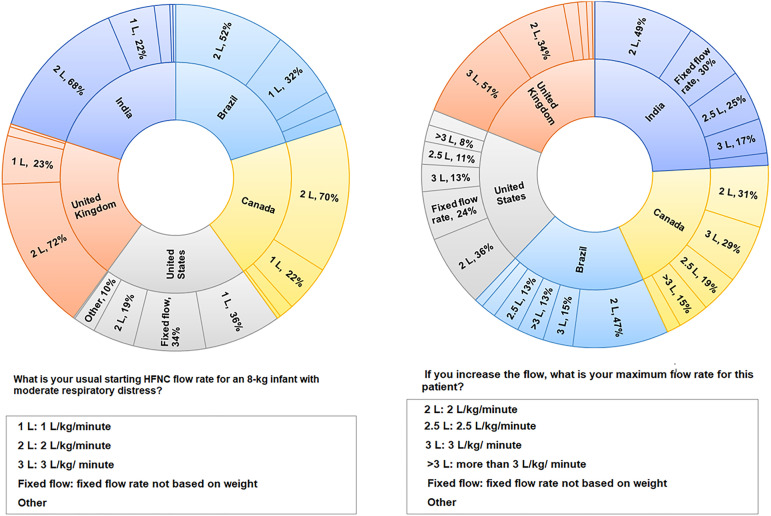
Proportions of responses for the clinical scenario of a 4-month-old child with respiratory distress, with need of high flow nasal cannula. HFNC - high flow nasal cannula.

A previously healthy 4-month-old infant (8kg weight) was admitted to the pediatric ICU with moderate respiratory distress due to bronchiolitis/pneumonia. A plan to initiate HFNC therapy as the primary therapy for respiratory distress is developed, and questions about the initial and maximum flow rates were asked. Most respondents in all groups agreed to start HFNC at 1 - 2L/kg/minute, but the proportions differed between the cohort from Brazil and the cohorts from the United Kingdom (p = 0.009), India (p = 0.02), Canada (p = 0.03), and the United States (p = 0.00). Most of those surveyed also considered increasing the maximum flow rate from 2 - 3L/kg/minute, although the proportions differed between the cohort in Brazil and the cohorts in the United Kingdom (p = 0.000) and the United States (p = 0.03). Fixed starting flows ranged from 2L/minute to 12L/minute. The responses regarding the initial and maximum flow rates are shown in figure 3.

When respiratory distress does not improve despite HFNC therapy at the maximum flow rate, 82% of the respondents in Brazil reported that they would consider a trial of NIV (e.g., continuous positive airway pressure - CPAP, bilevel NIV) for their patients before escalating to endotracheal intubation; this proportion was similar to that of the cohort in India, which was 76.8% (p = 0.2), whereas 93% of the respondents in the United Kingdom (p = 0.03), 88% in the United States (p = 0.42), and 91.5% in Canada (p = 0.14) reported that they would consider this treatment.

#### Case 2

**Figure 4:**
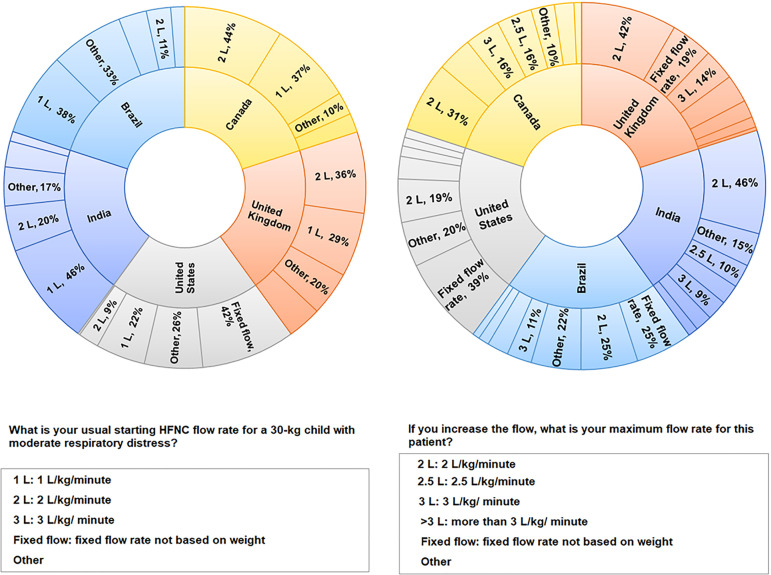
Proportions of answers for the clinical scenario of a 10-year-old child with respiratory distress, with need of high flow nasal cannula. HFNC - high flow nasal cannula.

A previously healthy 10-year-old child (30kg weight) is admitted to the pediatric ICU with moderate respiratory distress due to pneumonia. A plan to initiate HFNC therapy as the primary therapy for respiratory distress is developed, and the responses regarding initial and maximum flow rates are shown in figure 4. The proportions of responses for the starting flow rate for a child with a weight of 30kg were similar between Brazil and India (p = 0.06) but different between Brazil and each of the other countries. A significant proportion of respondents preferred 1L/kg/minute to start in all the groups. Fixed starting flows ranged from 8L/minute to 50L/minute. Other responses included intermediate values of 1.5L/Kg/minute. Regarding increasing the flow, the proportions of responses were similar between Brazil and the United States but different between Brazil and each of the other countries. Fixed increasing flows ranged from 20L/minute to 50L/minute.

When respiratory distress does not improve despite HFNC therapy at a maximum flow rate for the child, 81.3% of the respondents in Brazil reported that they would consider a trial of NIV for the patient before endotracheal intubation *versus* 90.8% in the United Kingdom (p = 0.05), 96.2% in the United States (p < 0.001), 96.6% in Canada (p < 0.001), and 76.8% in India (p = 0.56).

The preferred strategy for weaning HFNCs for patients with a primary respiratory disease was weaning the fraction of inspired oxygen (FiO_2_) to a specific value (most frequently 0.4) and subsequently weaning the flow rate.

When providing inhaled bronchodilators to a patient who received HFNC therapy, most physicians in Brazil reported that they preferred metered-dose inhalers with spacers with or without removing the HFNC(68.3%). In the other countries, the preference was for nebulization through the HFNC system with a special nebulizer inline, such as a vibrating mesh nebulizer, or an ordinary, noninline nebulizer with or without removing the HFNC; this was preferred by 88.5% of respondents in the United Kingdom, 90.2% in India, and 79% in the United States.

The decision to use a nasogastric tube to decompress the stomach was not a consensus. It was reported as frequent by only 7.4% of the respondents in Brazil and by 42% in the United Kingdom (p < 0.001), 15% in the United States (p = 0.07), 30.6% in Canada (p < 0.001), and 22.4% in India (p = 0.002).

Most of the respondents would feed the patient receiving HFNC therapy via enteral tubes instead of giving food orally, in the United Kingdom (91.9% *versus* 9.1%, p < 0.001), and Canada (75.4% *versus* 24.6% p = 0.002). In Brazil, 55% would feed via enteral tubes *versus* 45% orally (p = 0.07); in the United States, 49.6% choose enteral tubes *versus* 45.6% orally (p = 0.7), and 4.8% would keep no feed enteral or orally. In India, 67.2% choose enteral tubes *versus* 32.8% oral feeding (p = 0.07).

Only 6.5% of physicians in Brazil responded that they frequently used sedatives for patients on HFNC therapy, while 1.6% of respondents in the United Kingdom, India, and the US; and 1.8% in Canada provided this response. The use of sedatives was reported as occasional or infrequent by 74% of respondents in Brazil, 84% in the United Kingdom, 81.7% in the United States, 82.5% in Canada and 65.6% in India. The proportions of respondents that answered “never use sedation” were similar for Brazil (19.4%), the United Kingdom (14.5%, p = 0.36), the United States (16.7%, p = 0.62), and Canada (15.8%, p = 0.5) but different for India (32.8%, p = 0.03).

Table 3 shows the responses regarding the perception about clinical practices, cost-effectiveness and the occurrence of complications when comparing HFNC and CPAP. Respondents were asked to rank the three most important outcomes to be studied in future randomized trials comparing the effects of HFNC therapy and CPAP in pediatric patients with respiratory distress: a score was computed by attributing 3 points for first choice, 2 points for second choice and 1 point for third choice. The rate of endotracheal intubation was the most important outcome according to respondents in all countries, followed by the rate of failure (i.e., need for other modes of NIV or invasive ventilation) according to respondents in all the other countries but not in Brazil, where the pediatric ICU length of stay was the second most important outcome. As the third most important outcome, all respondents chose the length of mechanical ventilatory support, including HFNC except those in India, who chose patient comfort.

**Table 3:** Perception of the effectiveness and safety of high-flow nasal cannula when compared to continuous positive airway pressure

	Brazil	United Kingdom	United States	Canada	India
Clinical effectiveness					
Superior to CPAP	45.5	6.5	8.7	19.0	33.0
The same as CPAP	27.7	11.3	18.0	14.0	22.0
Inferior to CPAP	9.9	45.0	36.0	44.0	16.0
I do not know	11.9	21.0	20.5	15.8	20.6
p value for multiple proportions		0	0.00	0.00	0.14
Cost effectiveness					
Superior to CPAP	44.0	29.5	18.3	33.3	36.5
The same as CPAP	11.8	13.0	12.7	19.3	17.5
Inferior to CPAP	24.5	21.3	0	8.8	28.6
I do not know	19.6	24.6	65.1	36.8	15.9
p value for multiple proportions		0.38	0.00	0.001	0.47
Complications (fewer complications)					
Superior to CPAP	71.6	59.7	40.0	61.4	61.4
The same as CPAP	9.8	22.6	33.0	22.8	11.0
Inferior to CPAP	14.7%	3.0	2.4	5.3	12.7
I do not know	3.9	9.7	21.3	10.5	9.5
p value for multiple proportions		0.0	0.0	0.003	0.4

CPAP - continuous positive airway pressure. Note: the sum is not 100%, as some questions were not answered. p values refer to the proportions of responses. Results expressed as % if not indicated in a different way.

## DISCUSSION

The most contrasting finding in this study was that only 63.8% of the Brazilian responders had access to HFNCs, in contrast with 95% in the United Kingdom, 96.6% in the United States, 96.8% in Canada, and 84.7% in India (p < 0.001). This could be due to the late approval of the cannula in Brazil (late 2015) or to limited resources. Additionally, the use of HFNCs in general wards was unusual in Brazil (3%) and India (17.6%) but common in HICs. HFNC use was also reported in emergency departments and high-dependency care or pediatric ICU step-down units. HFNC therapy is a promising ventilatory support therapy for diseases that are frequent causes of pediatric ICU admissions, such as bronchiolitis. For this condition, it has been demonstrated to be cost-effective and less expensive than other modalities of treatment, but most of the data available are from studies in HICs.^(10)^ Implementation of HFNC therapy in limited-resource settings is feasible but poses technical challenges, not only due to the cost but also to the increased workload.^(11)^ Limited data suggest that the use of HFNC therapy in the pediatric general ward can reduce the demand for pediatric ICU beds in limited-resource settings.^(12)^

Brazil and India are middle-income countries (MICs), and the other countries in this study were HICs.^(13)^ Our survey shows a disparity in access to new technologies, as only 24.4% of the Brazilian physicians surveyed had access to extracorporeal membrane oxygenation (ECMO), 53.3%% had access to HFOV, and 63.8% had access to HFNCs. Although not universal, our findings suggest that HFNC therapy is now widely used in pediatric ICUs in MICs for various clinical situations, with bronchiolitis being the most frequent, but that its use is still more infrequent in MICs than in HICs. Most physicians agreed that respiratory distress and increased work of breathing are the most common situations in which HFNC therapy should be applied, followed by hypoxia. It is noteworthy that the proportion of respondents who reported that there is no protocol for HFNC therapy was 30.7% in Brazil and even lower in India (16.5%).

In Brazil, physicians are responsible for the implementation or weaning of HFNC together with respiratory therapists in the majority of cases, but only 5.5% of the respondents also reported that pediatric ICU trainees are responsible for these decisions. Interestingly, trainees in Brazil had a lack of autonomy in deciding to start HFNC therapy, a therapy that is not equivalent to extubating a patient, for example. In the HICs, there was more freedom for trainees, perhaps because only half of the units were university-based hospitals, where training must prepare learners for unsupervised practice.^(14)^ Additionally, 30% of the respondents did not know the number of admissions to their pediatric ICUs in Brazil; this may be related to the fact that many physicians work in two or three institutions to make a living in the country.^(15)^

In the two clinical scenarios involving children of different ages, we aimed to evaluate practices concerning the initial and maximal flows and strategies for weaning patients from HFNC therapy. One of the questions that remains unanswered when applying this therapy is how we should “dose” it. Pediatricians are trained to use weight-based dosing when prescribing drugs or setting the tidal volume on a mechanical ventilator.^(16)^ For the smallest child (weight of 8 kg), most of the respondents in all groups agreed to start HFNC therapy at 1 - 2L/kg/minute with a maximum flow rate from 2 - 3L/kg/minute. This seems to be the best practice according to the available evidence. Weiler et al. used esophageal manometry to calculate the pressure-rate product, a well-established surrogate for the effort of breathing.^(6)^ They found that the effort was sequentially reduced as HFNC flow rates were increased from 0.5L/kg/minute to 1.0L/kg/minute to 1.5L/kg/minute, but that the effect generally plateaued between 1.5L/kg/minute and 2.0L/kg/minute. Most benefits were seen in children that weighed ≤ 8kg. For the child with a weight of 30kg, slightly more than half of the respondents in the HICs but of only 40% in the MICs chose to start HFNC therapy at 1 - 2L/kg/minute. It is a common practice to start empirically with a set volume of 25 - 40L/minute for children aged 6 - 12 years, but there is a recommendation from manufacturers to change to an adult cannula for children weighing 25kg or greater, which favors an adult approach - a fixed rate of 20, 40 or 50L/minute.^(17)^ Initial flow rates of 50L/minute have been reported in prospective studies of critically ill adults and may be reasonable for adult-sized children and adolescents.^(18)^

Evidence for the use of gastric or enteral tubes for gastric decompression is lacking. Positive pressure in CPAP can distend the esophagus and decrease the esophageal sphincter pressure, leading to increased reflux, but it is unclear whether HFNC therapy causes a similar effect.^(19)^ Sochet et al. observed only one episode of aspiration-related respiratory failure among 132 children with bronchiolitis and receiving HFNC support, and oral nutrition was tolerated across a range of HFNC flow and respiratory rates.^(20)^ This study suggests that there is no evidence for withholding oral nutrition in these children, which is in line with the responses of the majority of the clinicians in this survey.

Most Brazilian doctors thought that HFNC therapy was clinically superior to or as efficient as CPAP, and the proportion was comparable to that of the respondents from India. The majority of respondents in the HICs thought that its clinical effect was inferior to or the same as that of CPAP. High-flow nasal cannula was also considered superior or the same as CPAP regarding cost-effectiveness and the occurrence of complications according to Brazilian respondents. These responses may reflect the lack of familiarity with or the availability of HFNCs and bilevel positive airway pressure (BIPAP) machines. While the use of HFNC therapy is increasing throughout the world, its efficacy and superiority to other forms of respiratory support is not completely established.^(21)^These data suggest that there is an opportunity for the advancement of HFNC therapy, research on HFNC therapy, and improving the quality of HFNC treatment in Brazilian pediatric ICUs.

Our study has several limitations. The relatively small sample size and demographic differences among Brazilian responders may have biased the results. Only members of the AMIB responded to the survey, and they may not be representative of Brazilian intensivists as a whole. The study was a post hoc analysis of a previously published survey, the original intent of which was not to ascertain if differences in practice related to HFNC were related to a country’s economic and social indicators.

## CONCLUSION

The availability of high-flow nasal cannulas in Brazil is still not widespread according to the respondents of this survey. There are some divergences in practices between Brazilian intensivists and their colleagues abroad, mainly in processes and decision-making about starting and weaning support with a high-flow nasal cannula. Future research should address the best practices on how to use a high-flow nasal cannula.

## References

[1] Coletti KD, Bagdure DN, Walker LK, Remy KE, Custer JW (2017). High-flow nasal cannula utilization in pediatric critical
care. Respir Care.

[2] Dysart K, Miller TL, Wolfson MR, Shaffer TH (2009). Research in high flow therapy: mechanisms of
action. Respir Med.

[3] Colleti J, Longui TE, Carvalho WB (2018). High-flow nasal cannula post-tracheal extubation in a child with
upper airway obstruction: case report. Rev Paul Pediatr.

[4] Rubin S, Ghuman A, Deakers T, Khemani R, Ross P, Newth CJ (2014). Effort of breathing in children receiving high-flow nasal
cannula. Pediatr Crit Care Med.

[5] Hutchings FA, Hilliard TN, Davis PJ (2015). Heated humidified high-flow nasal cannula therapy in
children. Arch Dis Child.

[6] Weiler T, Kamerkar A, Hotz J, Ross PA, Newth CJ, Khemani RG (2017). The relationship between high flow nasal cannula flow rate and
effort of breathing in children. J Pediatr..

[7] Pham TM, O'Malley L, Mayfield S, Martin S, Schibler A (2015). The effect of high flow nasal cannula therapy on the work of
breathing in infants with bronchiolitis. Pediatr Pulmonol.

[8] Saslow JG, Aghai ZH, Nakhla TA, Hart JJ, Lawrysh R, Stahl GE (2006). Work of breathing using high-flow nasal cannula in preterm
infants. J Perinatol.

[9] Kawaguchi A, Garros D, Joffe A, DeCaen A, Thomas NJ, Schibler A (2020). Variation in practice related to the use of high flow nasal
cannula in critically ill children. Pediatr Crit Care Med.

[10] Heikkilä P, Forma L, Korppi M (2016). High-flow oxygen therapy is more cost-effective for bronchiolitis
than standard treatment-A decision-tree analysis. Pediatr Pulmonol.

[11] Von Saint André-Von Arnim AO, Okeyo B, Cook N, Steere M, Roberts J, Howard CR (2019). Feasibility of high-flow nasal cannula implementation for
children with acute lower respiratory tract disease in rural
Kenya. Paediatr Int Child Health.

[12] Hoffman E, Reichmuth KL, Cooke ML (2019). A review of the use of high-flow nasal cannula oxygen therapy in
hospitalised children at a regional hospital in the Cape Town Metro, South
Africa. S Afr Med J.

[13] The Worl Bank World Bank Country and Lending Groups - World Bank Data Help
Desk.

[14] Schumacher DJ, Bria C, Frohna JG (2013). The quest toward unsupervised practice: promoting autonomy, not
independence. JAMA.

[15] Andrade-Nascimento M, Barros DS, Nascimento CL (2013). 1953 - Professional burnout syndrome among intensive care
physicians in Salvador, Brazil. Eur Psychiatry.

[16] Shein SL, Slain KN, Rotta AT (2017). High flow nasal cannula flow rates: new data worth the
weight. J Pediatr.

[17] Slain KN, Shein SL, Rotta AT (2017). The use of high-flow nasal cannula in the pediatric emergency
department. J Pediatr (Rio J).

[18] Frat JP, Thille AW, Mercat A, Girault C, Ragot S, Perbet S, Prat G, Boulain T, Morawiec E, Cottereau A, Devaquet J, Nseir S, Razazi K, Mira JP, Argaud L, Chakarian JC, Ricard JD, Wittebole X, Chevalier S, Herbland A, Fartoukh M, Constantin JM, Tonnelier JM, Pierrot M, Mathonnet A, Béduneau G, Delétage-Métreau C, Richard JC, Brochard L, Robert R, FLORALI Study Group, REVA Network (2015). High-flow oxygen through nasal cannula in acute hypoxemic
respiratory failure. N Engl J Med.

[19] Parlar-Chun R, Lafferty-Prather M, Gonzalez V, Pedroza C, Gourishankar A (2019). Protocol: randomised trial to compare nasoduodenal tube and
nasogastric tube feeding in infants with bronchiolitis on high-flow nasal
cannula; Bronchiolitis and High-flow nasal cannula with Enteral Tube feeding
Randomised (BHETR) trial. BMJ Open.

[20] Sochet AA, McGee JA, October TW (2017). Oral nutrition in children with bronchiolitis on high-flow nasal
cannula is well tolerated. Hosp Pediatr.

[21] Colleti J, Azevedo R, Araujo O, Carvalho WB (2020). High-flow nasal cannula as a post-extubation respiratory support
strategy in preterm infants: a systematic review and
meta-analysis. J Pediatr (Rio J).

